# Southern Climate

**Published:** 1856-11

**Authors:** 


### SOUTHERN CLIMATE.

It is a standing direction to go to a warmer climate in threatened or actual consumption.

Warm weather takes away the energies of the healthiest among us, and the universal experience of physicians and patients is that it debilitates consumptives greatly. If warm weather at home debilitates, how can it fail to debilitate when away from home?

The chemist knows that there is more nutriment in a pint of cold air than there is in a pint of warm air, because it is more condensed. It is a conceded point in consumption that the larger the quantity of air the patient can consume the greater are his chances of recovery. The fewer lungs he has, the more reason there is that he should consume the most concentrated and purest air there is. Besides the rarefaction of a Southern atmosphere, it must necessarily be loaded with vapor, and partly so with miasm, the fruitful cause of violent diseases. These suggestions will bear study.

In some liver affections, the person loses flesh, pales away and with more or less cough, the friends become alarmed, and fear that he is going in a decline. He is sent South, and relief from business cares, change of air and scene, and food, and habits of life, soon restore him, and he returns home a well man. And, like the drawing of the highest prize in a lottery, the one success sends thousands on the same errand, to meet with a hopeless failure, for, in the first case, it was a disease of the liver, which readily yields to the remedy, because it was applicable, but in the other case it was a disease of the lungs, which, if in the advanced stages is rendered more speedily and certainly fatal. In forming consumption, out-door activities abate all the symptoms, and the patient hurries home, feeling well, but not having kept up these activities long enough, the tendency to disease of that kind has not been fully broken up, a habit of health has not been established, and slight causes bring a return of the symptoms, and you see such persons going to the South every winter, until the system loses its power of recuperation from these fitful efforts, and the final result is "died of consumption" the very case that had been noised about a few years before as having been cured of consumption by going to the South. Thus it is that the gross error is kept alive, and is almost a universal belief. A few years ago, a gentleman of note sufficient to have his movements chronicled in the papers, left home for a throat affection, with a view to spending a winter in the South. Circumstances led him to call on the author for advice, in this city, and he returned home, and is in good health to this day. But shortly after his resumption of professional duty it was announced in the papers that this gentleman's visit to the South had fully restored him.

It is a significant fact, that the British government sends its consumptive soldiers from its Southern stations towards the North. The reader is referred for statistical statements on this subject to *Bronchitis and Kindred Diseases*, 8th edition.

Very much has been said of Italian skies, and of the South of France, but the simple fact that the natives of these localities do not reach the average of human life which prevails in England and more Northern latitudes, is an unanswerable argument against the salubrity of those far-famed localities.

